# Identification of common mechanisms and biomarkers for dermatomyositis and atherosclerosis based on bioinformatics analysis

**DOI:** 10.1111/srt.13808

**Published:** 2024-06-20

**Authors:** Yirong Ma, Junyu Lai, Qiang Wan, Zhengtao Chen, Liqiang Sun, Qinhe Zhang, Chengyan Guan, Qiming Li, Jianguang Wu

**Affiliations:** ^1^ Jiangxi University of Traditional Chinese Medicine Nanchang Jiangxi China; ^2^ Department of cardiovascular Affiliated Hospital of Jiangxi University of Traditional Chinese Medicine Nanchang Jiangxi China

**Keywords:** atherosclerosis, bioinformatics analysis, dermatomyositis, gene, expression, GEO database, immune infiltration analysis

## Abstract

**Background:**

Dermatomyositis (DM) manifests as an autoimmune and inflammatory condition, clinically characterized by subacute progressive proximal muscle weakness, rashes or both along with extramuscular manifestations. Literature indicates that DM shares common risk factors with atherosclerosis (AS), and they often co‐occur, yet the etiology and pathogenesis remain to be fully elucidated. This investigation aims to utilize bioinformatics methods to clarify the crucial genes and pathways that influence the pathophysiology of both DM and AS.

**Method:**

Microarray datasets for DM (GSE128470, GSE1551, GSE143323) and AS (GSE100927, GSE28829, GSE43292) were retrieved from the Gene Expression Omnibus (GEO) database. The weighted gene co‐expression network analysis (WGCNA) was used to reveal their co‐expressed modules. Differentially expression genes (DEGs) were identified using the “limma” package in R software, and the functions of common DEGs were determined by functional enrichment analysis. A protein‐protein interaction (PPI) network was established using the STRING database, with central genes evaluated by the cytoHubba plugin, and validated through external datasets. Immune infiltration analysis of the hub genes was conducted using the CIBERSORT method, along with Gene Set Enrichment Analysis (GSEA). Finally, the NetworkAnalyst platform was employed to examine the transcription factors (TFs) responsible for regulating pivotal crosstalk genes.

**Results:**

Utilizing WGCNA analysis, a total of 271 overlapping genes were pinpointed. Subsequent DEG analysis revealed 34 genes that are commonly found in both DM and AS, including 31 upregulated genes and 3 downregulated genes. The Degree Centrality algorithm was applied separately to the WGCNA and DEG collections to select the 15 genes with the highest connectivity, and crossing the two gene sets yielded 3 hub genes (PTPRC, TYROBP, CXCR4). Validation with external datasets showed their diagnostic value for DM and AS. Analysis of immune infiltration indicates that lymphocytes and macrophages are significantly associated with the pathogenesis of DM and AS. Moreover, GSEA analysis suggested that the shared genes are enriched in various receptor interactions and multiple cytokines and receptor signaling pathways. We coupled the 3 hub genes with their respective predicted genes, identifying a potential key TF, CBFB, which interacts with all 3 hub genes.

**Conclusion:**

This research utilized comprehensive bioinformatics techniques to explore the shared pathogenesis of DM and AS. The three key genes, including PTPRC, TYROBP, and CXCR4, are related to the pathogenesis of DM and AS. The central genes and their correlations with immune cells may serve as potential diagnostic and therapeutic targets.

## 1 INTRODUCTION

Dermatomyositis (DM) represents a systemic autoimmune condition, marked by distinct rashes and muscle inflammation, frequently leading to muscle weakness.^1^ Typical features include periorbital rash (heliotrope rash), erythema over the extensor surfaces of joints, and perifascicular atrophy,^2^ which classify it as a subgroup of idiopathic inflammatory myopathies (IIM).^3^ The etiology and pathogenesis of DM have not been fully elucidated, with genetic predispositions, environmental stressors, immune and non‐immune triggering mechanisms, and abnormalities in interferon pathway signaling all being related to the susceptibility and onset of the disease.^4^ Atherosclerosis (AS) is the primary pathological process in cardiovascular disease, and has emerged as a major contributor to global morbidity and mortality.^5^ The Global Burden of Disease Study delineates a significant escalation in the prevalence of cardiovascular conditions, escalating from 271 million cases in 1990 to 523 million in 2019, reflecting an almost twofold increase;concurrently, mortality attributable to these diseases has witnessed a continuous upsurge, moving from 12.1 million in 1990 to 18.6 million in 2019.^6^ AS is characterized by the accumulation of fibro‐fatty lesions in the arterial wall and infiltration by immune cells such as macrophages, T‐cells, and mast cells, representing a chronic inflammatory disease with autoimmune components.^7^ It involves complex interactions between immune metabolic changes and oxidative stress. Extramuscular manifestations of DM include cardiac abnormalities, interstitial lung disease, and malignancies.^8^ A study in the United States revealed that one of the most significant causes of death among DM cases, based on data from 1981 to 2020, was heart disease,^9^, ^10^ including coronary artery atherosclerotic heart disease, heart failure, and myocardial infarction. To date, the largest study, based on the United States National Database, included 50,322 DM patients and found that 20% of DM patients had concurrent atherosclerotic cardiovascular diseases, with an overall hospital mortality rate of 5.7%. Multifactorial analysis showed that the mortality rate of DM patients with cardiovascular disease was twice that of DM patients without cardiovascular disease and 1.98 times that of the general population with cardiovascular disease.^11^ DM, especially in its severe forms, can aggravate AS, suggesting that DM acts as an autonomous risk factor for these conditions.

Although the precise nature of the connection between DM and AS remains elusive, it is apparent that both conditions are significantly influenced by inflammatory responses and aberrant immune system activation. The pathogenesis of AS primarily involves the subendothelial deposition of low‐density lipoprotein (LDL), triggering local inflammatory responses.^12^ Subsequently, macrophages that engulf LDL form foam cells, further exacerbating inflammation leading to endothelial damage and stiffening of the vascular wall.^13^ Patients with DM exhibit significant inflammatory cell infiltration in muscle tissues and skin, such as T‐cells and macrophages, and an increase in inflammatory mediators (such as cytokines), which is currently believed to be caused by the immune system's erroneous attack on muscle and skin tissues, leading to inflammation and tissue damage.^14^ Furthermore, in the study of DM and AS, some biomarkers, such as C‐reactive protein (CRP), interleukins (ILs), and tumor necrosis factor α (TNF‐α), have shown potential value in monitoring disease activity and inflammation levels. These indicators may be elevated in both diseases, suggesting that inflammation plays a central role in both conditions.^15^, ^16^, ^17^, ^18^ Therefore, identifying the infiltration of immune cells and the corresponding inflammatory markers holds significant diagnostic value for patients with DM who also suffer from AS. This is of paramount importance in circumventing grave cardiovascular outcomes.

**FIGURE 1:**
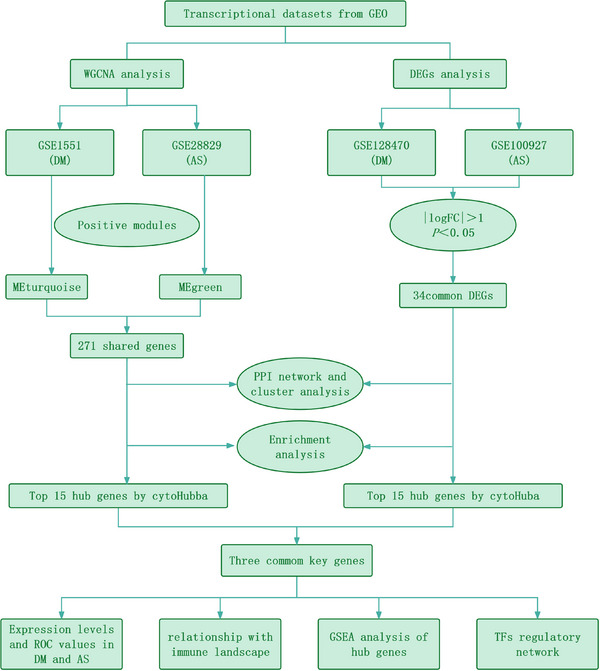
The flowchart for bioinformatic analysis.

Although numerous studies have confirmed a link between DM and AS, their common etiology and pathogenesis remain unclear. Therefore, It is of paramount importance to investigate and clarify the molecular pathways common to DM and AS emerges as a critical and immediate requirement, enabling the development of targeted clinical interventions. Through the examination of the genetic foundations and mutual molecular pathways linking DM and AS, a more profound comprehension of the mechanisms contributing to the co‐occurrence can be achieved. The objective of this investigation is to pinpoint co‐expression modules that are linked with DM and AS by analyzing gene expression profiles sourced from public databases. Concurrently, we conducted both WGCNA and DEG analysis across multiple datasets to pinpoint genes common to both conditions. Following this, functional enrichment analysis was undertaken to investigate shared pathways. Moreover, through the utilization of PPI and cluster analysis, we pinpointed critical genes associated with both conditions and examined the link between these key genes and immune infiltration in DM and AS. Figure 1 illustrates the research methodology. The uncovering of these shared genetic markers and molecular pathways is to delve deeper into the common pathogenesis of DM and AS, with the ultimate goal of identifying novel therapeutic targets that could enhance the prognosis for individuals impacted by these diseases.

## 2 MATERIALS AND METHODS

### 2.1 GEO dataset download

Using the keywords “DM” and “AS,” Series matrix files and platform information of GSE128470, GSE1551, GSE143323, GSE100927, GSE28829 and GSE43292 were obtained from the National Center Biotechnology Information Gene Expression Omnibus (NCBI‐GEO) (https://www.ncbi.nlm.nih.gov/geo). In subsequent bioinformatics analysis, GSE1551 and GSE28829 were aligned to conduct WGCNA, while GSE128470 and GSE100927 were paired for DEG analysis, GSE143323 and GSE43292 were used as external datasets for validation.

### 2.2 Weighted gene coexpression network analysis

The methodology known as weighted gene co‐expression network analysis (WGCNA) facilitates the identification of gene modules with significant biological relevance and explores the connections between gene networks and diseases.^19^, ^20^ Consequently, the WGCNA package in R software (version 4.3.2) was employed to detect modules related to DM and AS within the datasets GSE1551 and GSE28829. The analysis was confined to approximately 5000 genes, given that the expression levels of most genes did not exhibit notable differences across samples. The “hclust” function in R was applied to remove a specific sample from the GSE1551 dataset during the hierarchical clustering analysis. Subsequently, the R function “pickSoftThreshold” was employed to determine the soft thresholding power *β*, which elevates co‐expression similarity for the adjacency calculation. Following this, the adjacency was converted into a topological overlap matrix (TOM), which was utilized to assess the connectivity within the gene network. The soft threshold *β* was set to 2 for DM, and to 12 for AS (Supplementary Materials 1 and 2). Additionally, we employed hierarchical clustering to develop a dendrogram that organizes gene expressions into various modules. These modules were then individually marked with different shades of color, establishing a baseline color intensity of 60. To encapsulate the expression trends within each module succinctly, Module Eigengenes (MEs) were deployed. Following this, evaluation of the relationships between the MEs and clinical attributes was conducted through correlation analysis. Priority is given to modules closely associated with clinical features for subsequent research protocols.

### 2.3 Identification of cross‐genes between DM and AS through WGCNA

The selection of modules was informed by their robust positive association with DM or AS, anchored on the correlation metrics between the modules and the diseases, alongside the *p*‐values associated with the module‐specific genes to phenotypic characteristics. Utilizing the Jvenn online tool,^21^ 271 intersecting genes were obtained from these modules.

### 2.4 Differential gene enrichment analysis

The limma package,^22^ within the R software environment (Version 4.3.2), was deployed to identify differentially expressed genes (DEGs) between patients with DM and healthy controls, as well as between patients with arteriosclerosis and healthy controls, as referenced in studies.^23^, ^24^, ^25^, ^26^ DEGs were filtered based on the criteria of an absolute |log FC| > 1 and adjusted *p‐*value < 0.05

### 2.5 Functional enrichment analysis

In our study, the “clusterProfiler”packagewas used to perform Gene Ontology (GO) and Kyoto Encyclopedia of Genes and Genomes (KEGG) analyses on the shared genes This approach facilitated the identification of biological functions and pathways associated with the genes of interest. Significant pathways were identified with *p* < 0.05

### 2.6 PPI network construction and cluster analysis

We utilized the STRING database (http://string‐db.org) for retrieving interacting genes. The PPI network was visualized using Cytoscape (Version 3.9.1), with a minimum interaction score of >0.4. Subsequently, the MCODE plugin application was employed to isolate clusters exhibiting high connectivity, thereby partitioning the Protein‐Protein Interaction (PPI) network into multiple clusters. The criteria were as follows: degree cutoff = 2, node score cutoff = 0.2, k‐core = 2, and maximum depth = 100.

### 2.7 Validate hub genes

In our study, the cytoHubba plugin's degree centrality method was applied to pinpoint key genes within the PPI network that exhibited substantial interconnectivity. Utilizing the Jvenn online platform,^21^ an intersection was performed between the foremost 15 central genes delineated through WGCNA and the top 15 central genes identified from DEG analysis, culminating in the discovery of three genes central to both datasets. The expression levels of these pivotal shared genes were subsequently corroborated across four distinct datasets employing the “ggpubr” package in R. Additionally, the diagnostic potential of these shared hub genes was assessed by generating ROC curves, facilitated by the pROC package in R.

### 2.8 Immune infiltration analysis

We employed the CIBERSORT algorithm^27^ to evaluate the relative percentages and significance (*p*‐values) of 22 types of immune cells in the blood of DM and AS patients. Using this method, we transformed a gene expression dataset into a profile of diverse immune cell populations, leveraging the LM22 signature from the CIBERSORT website (http://CIBERSORT.stanford.edu/) for accurate identification. We focused only on results with *p*‐values ≤ 0.05, indicating significant differences in immune cell composition. The outcome is a matrix depicting various immune cell subpopulations, visualized through R packages “corrplot”, “ggplot2”,^28^ and “ggpubr” for a clear representation of the data.

### 2.9 Gene set enrichment analysis (GSEA)

The “clusterProfiler” package in R was employed to conduct single‐gene Gene Set Enrichment Analysis (GSEA) for DM and AS, subsequent to pinpointing central hub genes. Individuals diagnosed with DM or AS were stratified into high and low gene expression groups, using the median expression level of these hub genes as the threshold for categorization. GSEA facilitated the computation of enrichment scores for gene sets, thereby elucidating distinct functional phenotypes. Additionally, this method was applied to discern differences in biological pathways between the two expression groups. The c5.go.bp.v7.5.1.entrez.gmt gene set was used as a reference. *p*‐Value < 0.05, normalized enrichment score (NES) > 1, and false positive rate (FDR) *q*‐value < 0.05 were considered as significantly enriched gene sets. Enrichment plots highlighted the five most prominent activated and inhibited pathways associated with each hub gene in both diseases.

### 2.10 TF‐gene network of hub genes

The NetworkAnalyst tool (version 2019; https://www.networkanalyst.ca/) was utilized for mapping interactions among transcription factor (TF) genes and central genes. Moreover, the relationships between shared central genes and TFs were illustrated.

## 3 RESULTS

### 3.1 Identification of shared genetic features between DM and AS through WGCNA

**FIGURE 2:**
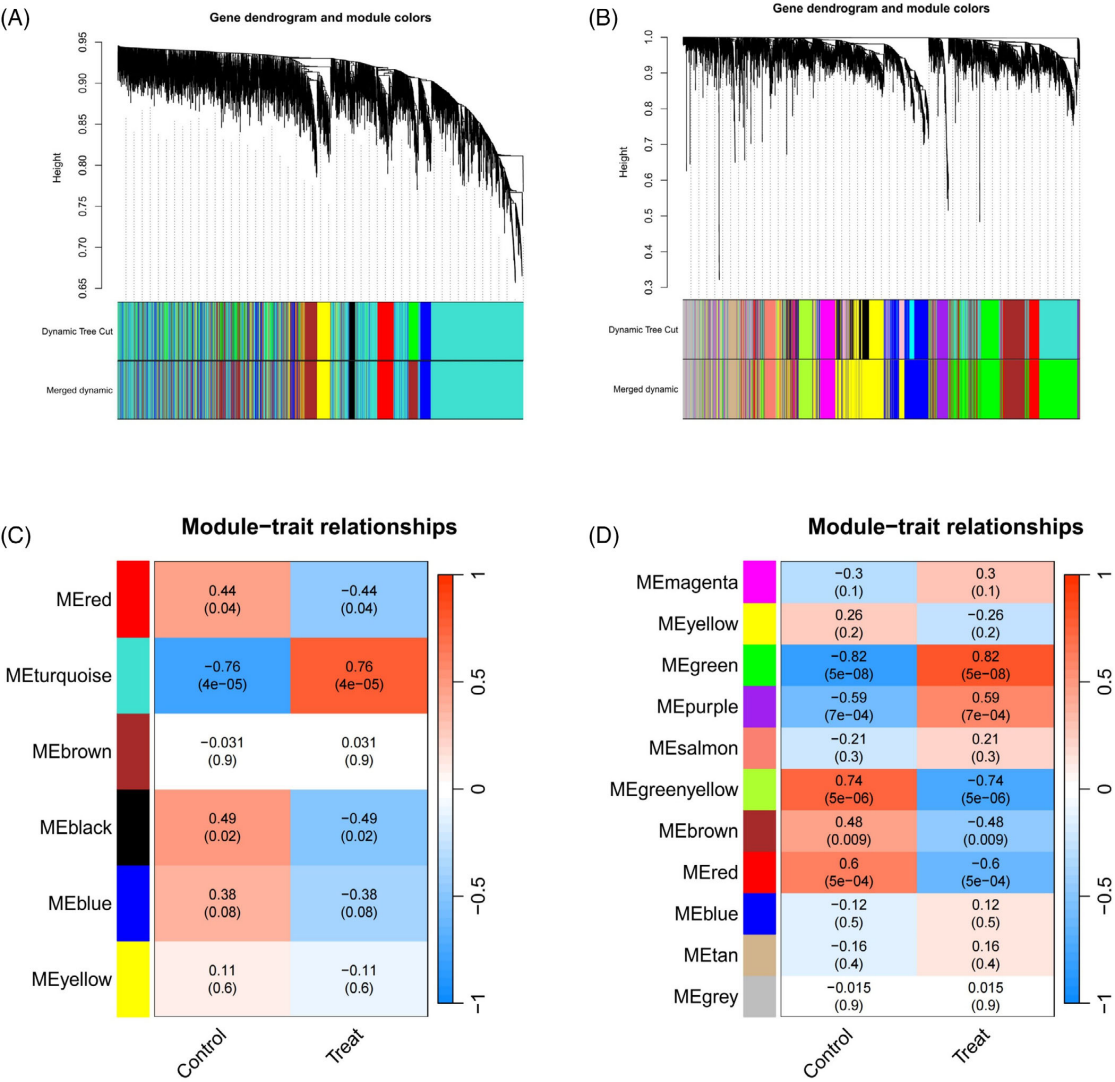
Screening of genes in the GSE143323 (DM) and GSE28829 (AS) datasets using the WGCNA algorithm. (A), (B) The Cluster dendrogram in GSE143323 (DM) and in GSE28829 (AS). (C), (D) Heatmap illustrating the module‐trait relationships in GSE143323 (DM) and GSE28829 (AS). AS, atherosclerosis; DM, dermatomyositis; WGCNA, weighted gene coexpression network analysis.

**FIGURE 3:**
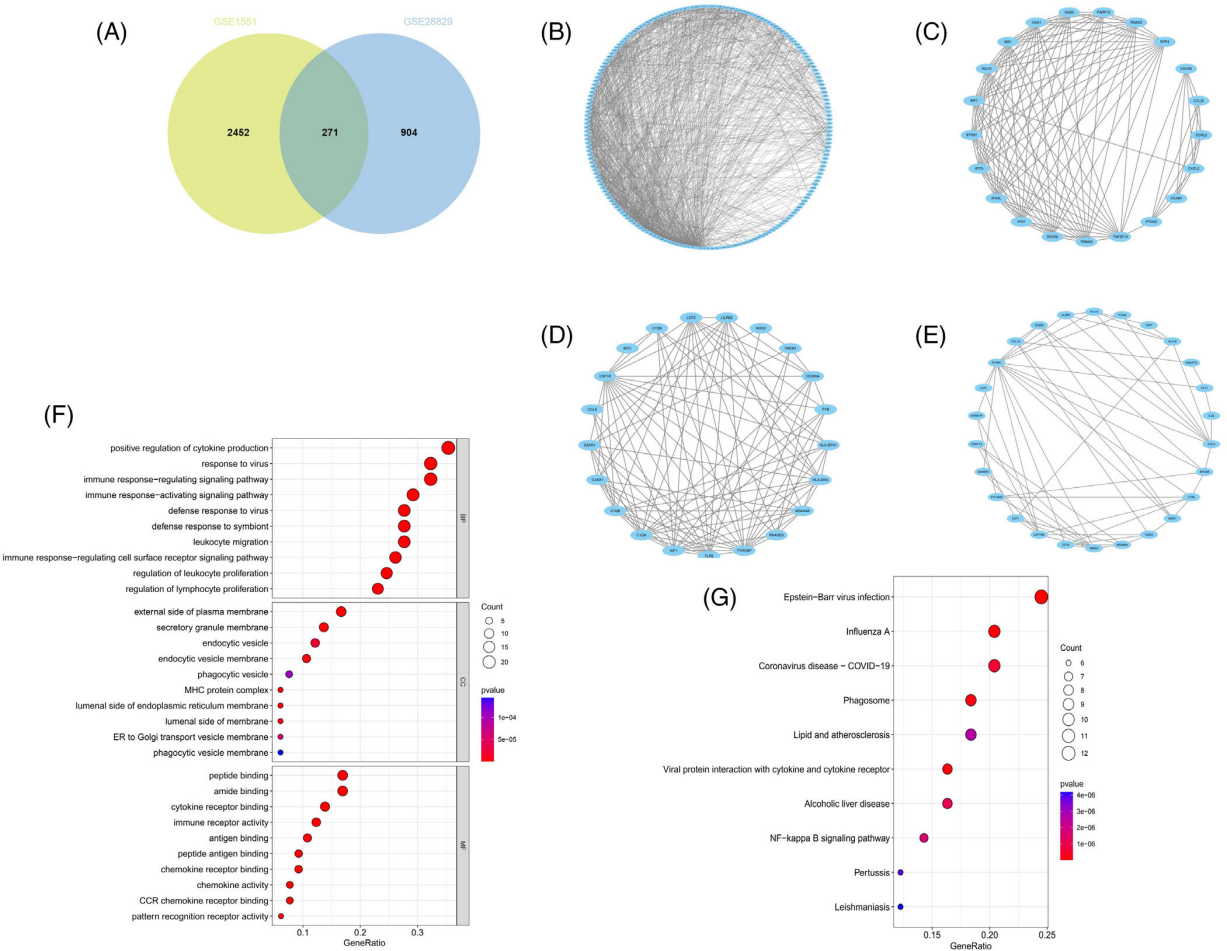
Venn plot of common genes between DM and AS and the PPI network of the intersecting genes. (A) The overlapped genes between the turquoise module in GSE1551 (DM) and the green module in GSE28829 (AS). (B)–(E) A PPI network for the 192 common genes and three clusters extracted using MCODE. (F) Bubble plot of GO enrichment analysis of three gene clusters. (G) Bubble plot of KEGG enrichment analysis of three gene clusters. AS, atherosclerosis; DM, dermatomyositis; GO, Gene Ontology; IPF, idiopathic pulmonary fibrosis; KEGG, Kyoto encyclopedia of genes and genomes; MCODE, minimal common oncology data elements; PPI, protein‐protein interaction.

Within the GSE1551 dataset, WGCNA discerned six distinct modules, each symbolized by a unique color. The association between these modules and specific diseases was evaluated via Spearman correlation coefficient heatmaps. The module labeled “MEturquoise” is particularly notable for its significant correlation with DM, which has led to its classification as a module related to DM (MEturquoise: *r* = 0.76, *p* = 4e‐05). In an analysis of the GSE28829 dataset, it was determined that among the 11 modules unearthed, the module known as “MEgreen” had the most pronounced correlation with AS (MEgreen: *r* = 0.82, *p* = 5e‐08) (Figures 2A–D). Intersection of DM and AS positively correlated gene modules (MEturquoise and MEgreen) (Supplementary Materials 3 and 4) resulted in 271 shared genes (Figure 3A). The PPI network for these genes was constructed using STRING, leading to a network of 192 nodes and 1128 links after isolating proteins were excluded (Figure 3B). The MCODE plugin identified three closely connected gene clusters (Figures 3C–E). Functional enrichment analysis of these three clusters highlighted involvement in immune and inflammatory responses, with GO pathways indicating significant enrichment in immune‐related biological processes and KEGG pathways underscoring the genes' roles in infectious diseases and immune regulation processes (Figures 3F and 3G). This analysis suggests DM and AS's central involvement in immune system modulation and inflammation.

### 3.2 Identification and analysis of DEGs

**FIGURE 4:**
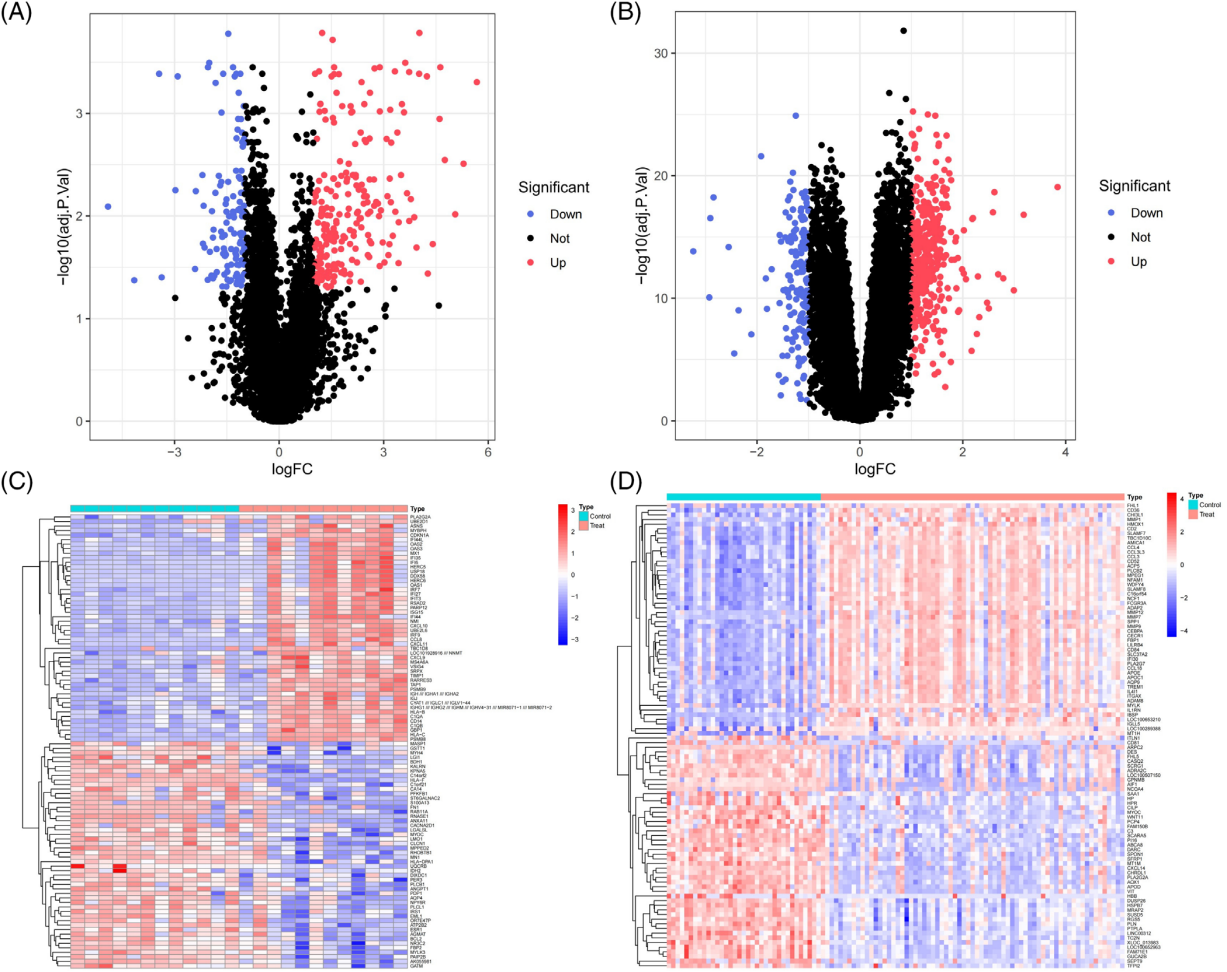
Volcano plot and Heatmap of the DEGs identified from GSE128470 and GSE100927. (A), (B) Volcano map of DEGs from GSE128470 and GSE100927. (C), (D) Heatmap of DEGs from GSE128470 and GSE100927. DEGs, differentially expression gene.

**FIGURE 5:**
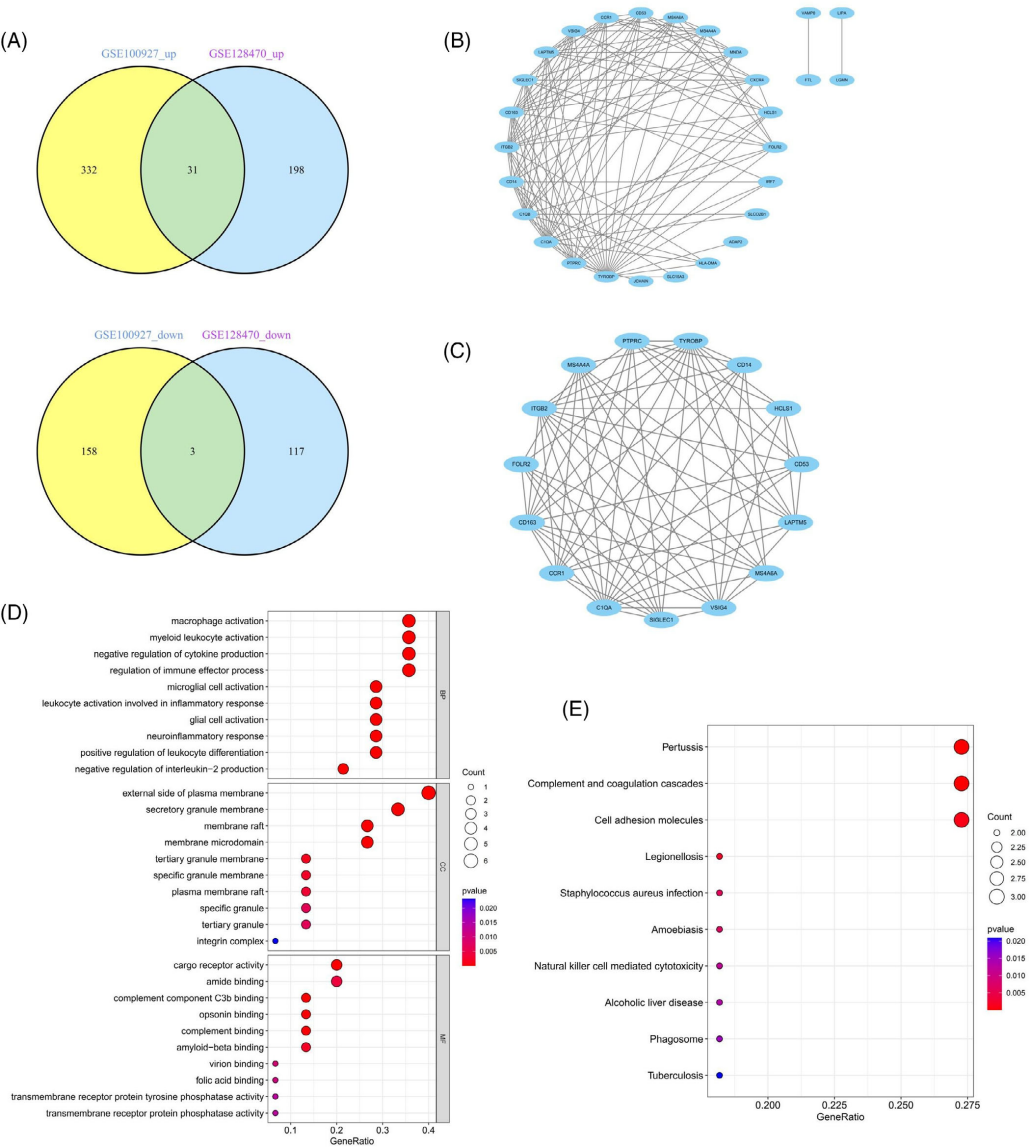
Verification and analysis of shared DEGs in DM and AS. (A) Venn diagram for shared DEGs in the GSE128470 and GSE100927 datasets. (B) The PPI network of the shared DEGs. (C) One cluster extracted by MCODE. (D) Bubble plot of GO enrichment analysis of common DEGs. (E) Bubble plot of KEGG enrichment analysis of common DEGs. AS, atherosclerosis; DEGs, differentially expression gene; DM, dermatomyositis; GO, Gene Ontology; IPF, idiopathic pulmonary fibrosis; KEGG, Kyoto encyclopedia of genes and genomes; MCODE, minimal common oncology data elements.

DEG analysis delineated 349 DEGs in the GSE128470 dataset, comprising 229 genes with increased expression and 120 with diminished expression. Similarly, in the GSE100927 dataset, 524 DEGs were discerned, including 363 genes exhibiting elevated expression levels and 161 demonstrating reduced expression levels. Visualization of these DEGs was accomplished through the employment of heatmaps and volcano plots, as depicted in (Figures 4A–D). A Venn diagram highlighted the intersection of these datasets, revealing 34 genes shared between them, with 31 displaying concurrent upregulation and 3 showing downregulation (Figure 5A). Furthermore, the construction of a PPI network for these shared genes yielded a network comprising 28 nodes and 121 links, following the removal of solitary genes (Figure 5B). An analysis using the MCODE plugin's algorithm facilitated the extraction of a significant cluster containing 15 nodes and 74 edges (Figure 5C). Subsequently, the GO analysis of these DEGs revealed that BP genes were mainly enriched in macrophage activation, negative regulation of cytokine production, regulation of immune effector processes, and activation of leukocytes and glial cells (Figure 5D). CC genes were enriched in the external side of the plasma membrane, secretory granule membrane, membrane rafts, and membrane microdomains. MF‐related genes were enriched in cargo receptor activity, amide binding, complement binding, and antigen binding pathways. KEGG analysis showed significant enrichment in pertussis, complement and coagulation cascades, cell adhesion molecules, Legionnaires' disease, and Staphylococcus aureus infection (Figure 5E). In alignment with the findings from the WGCNA, the processes of “antigen processing and presentation” along with “microbial infection” were once more found to be prominently enriched.

### 3.3 Selection and verification of common genes

**FIGURE 6:**
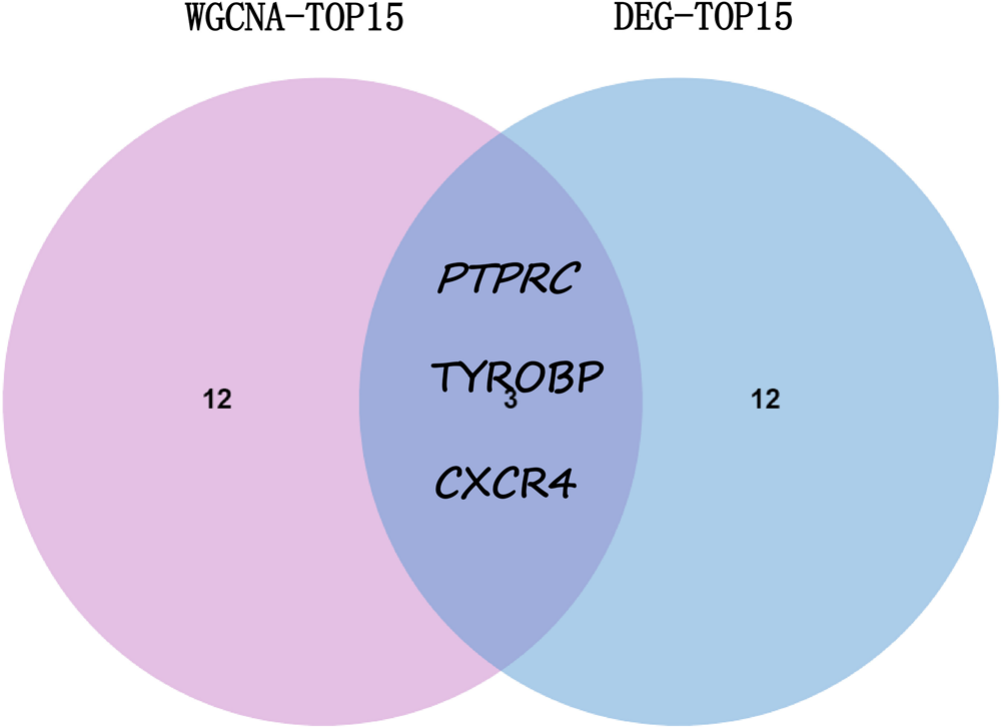
Screening out of common hub genes through the intersection of the top 15 genes derived from DEG and WGCNA. DEGs, differentially expressed genes; WGCNA, weighted gene coexpression network analysis.

**FIGURE 7:**
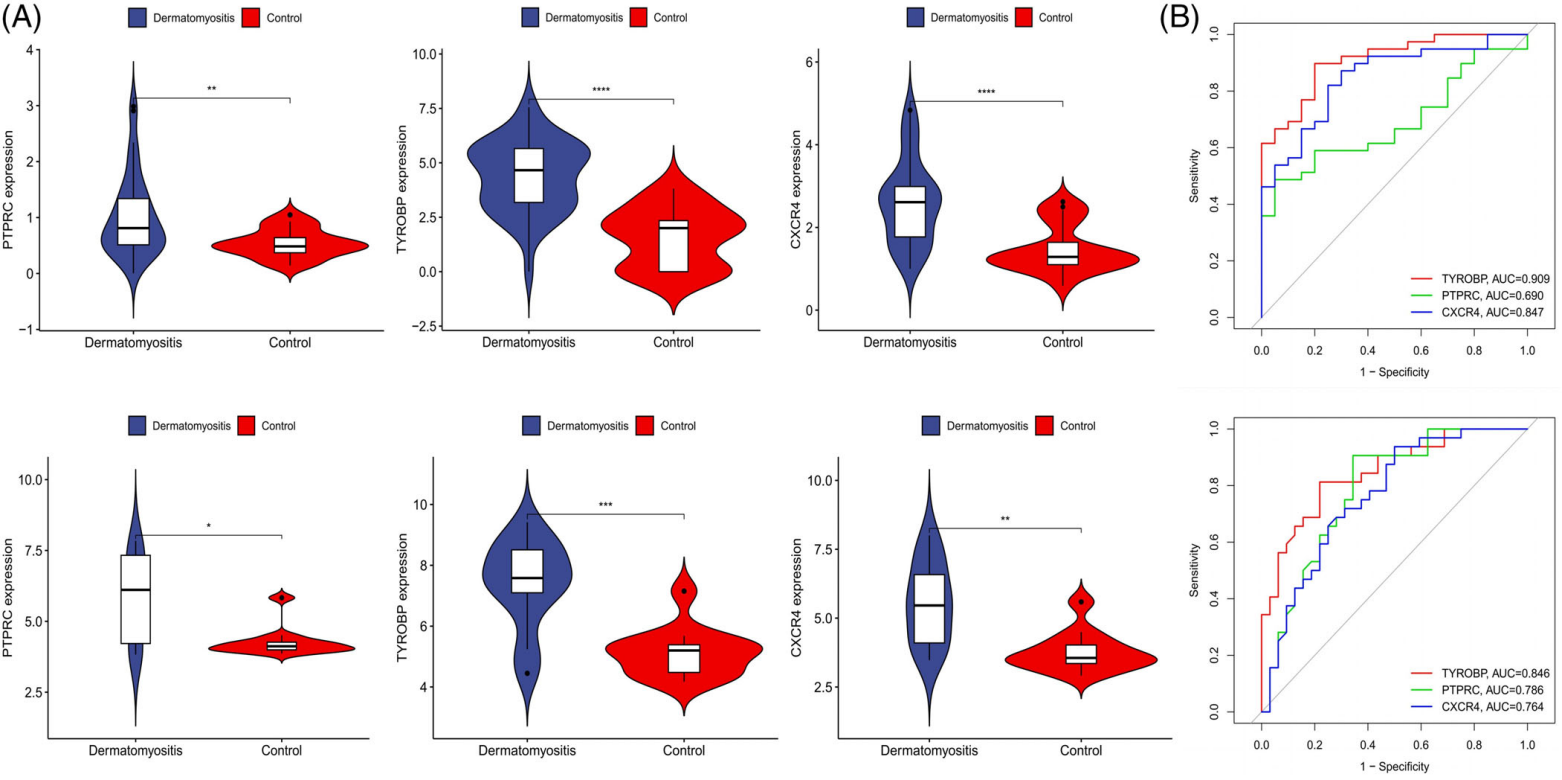
The expression levels and diagnostic efficacy of the common genes in DM datasets. (A) PTPRC, TYROBP and CXCR4 expression levels in two DM databases, with blue violin plots representing DM, red representing controls. Students’*t*‐test with *p* < 0.05 was used to determine statistical significance. ^*^ *p* < 0.05; ^**^ *p* < 0.01;^***^ *p* < 0.001; ^****^ *p* < 0.0001. (B) The ROC curves showing AUC values of PTPRC, TYROBP and CXCR4 in DM. DM, dermatomyositis; AUC, area under curve.

**FIGURE 8:**
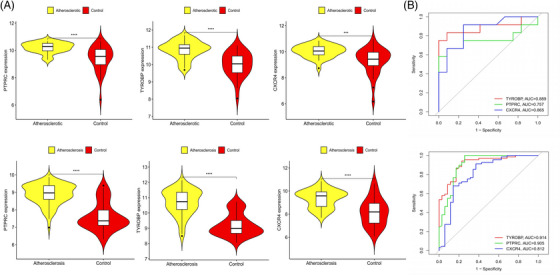
The expression levels and diagnostic efficacy of the common genes in AS datasets. (A) PTPRC, TYROBP and CXCR4 expression levels in two AS databases, with yellow violin plots representing AS, red representing controls. Students’*t*‐test with *p* < 0.05 was used to determine statistical significance. ^*^ *p *< 0.05; ^**^ *p *< 0.01;^***^ *p* < 0.001; ^****^ *p* < 0.0001. (B) The ROC curves showing AUC values of PTPRC, TYROBP and CXCR4 in AS. AS, Atherosclerosis; AUC, area under curve.

Common genes were pinpointed via PPI network analysis, employing the Cytoscape plugin cytoHubba.^29^ The Degree Centrality algorithm, which identifies each gene's adjacent nodes, indicating that the more adjacent nodes a gene has, the more likely it is to be a core gene in the regulatory network, identified the top 15 genes as potential hubs. Subsequently, three genes (PTPRC, TYROBP, CXCR4) were identified among the top 15 central genes in the WGCNA and DEG datasets (Figure 6). The expression levels of these genes were subsequently corroborated across four distinct datasets. Intriguingly, all three genes exhibited increased expression levels in conditions of DM and AS when contrasted with the control group (Figures 7A and 8A). Moreover, our analysis revealed that these three genes hold substantial diagnostic importance for both DM and AS. This was substantiated by the Area Under the Curve (AUC) metrics derived from the analysis, where GSE43292 and GSE100927 datasets yielded AUC values surpassing 0.75, thereby indicating a robust diagnostic capability for AS. Concurrently, the GSE143323 and GSE128470 datasets revealed AUC values primarily exceeding 0.8, underscoring their diagnostic relevance in DM scenarios, with TYROBP in particular showcasing an AUC value over 0.85 (Figures 7B and 8B), highlighting its significant diagnostic efficacy.

### 3.4 Immune cell infiltration and its correlations with hub genes

**FIGURE 9:**
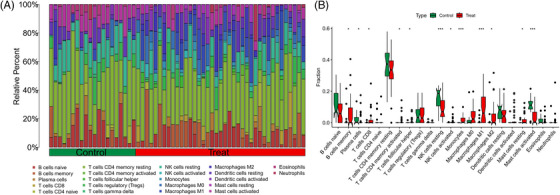
Immune infiltration analysis of DM. (A) Barplot of the 22 immune cells proportion. (B) Comparison of immune cell proportion between DM and controls (Willcoxon's test). ^*^ *p* < 0.05; ^**^ *p* < 0.01; ^***^ *p* < 0.001. DM, dermatomyositis.

**FIGURE 10:**
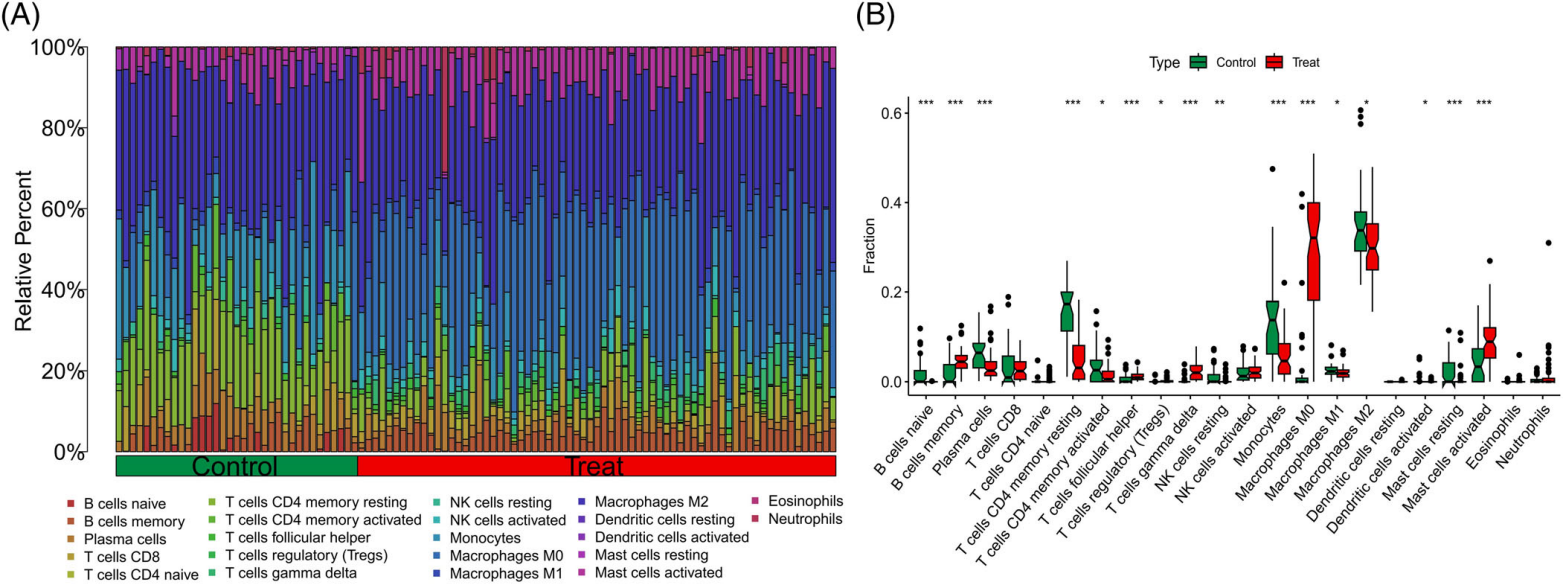
Immune infiltration analysis of AS. (A) Barplot of the 22 immune cells proportion. (B) Comparison of immune cell proportion between AS and controls (Willcoxon's test). ^*^ *p* < 0.05; ^**^ *p* < 0.01; ^***^ *p* < 0.001. AS, atherosclerosis.

**FIGURE 11:**
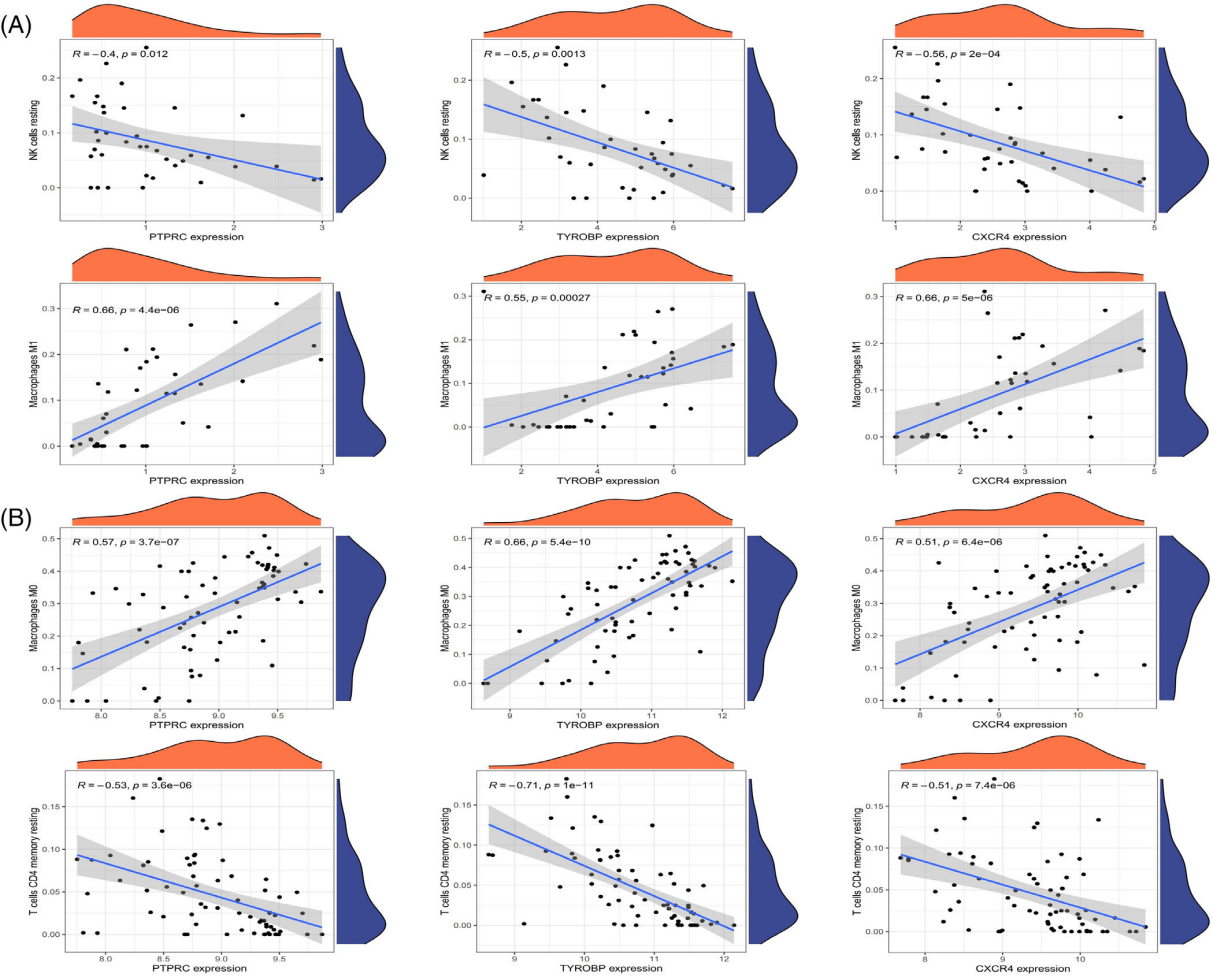
Correlation analysis between hub genes and immune cells. (A) Correlation analysis between hub genes and immune cells in DM. (B) Correlation analysis between hub genes and immune cells in AS. AS, atherosclerosis; DM, dermatomyositis.

Samples from GSE143323 and GSE100927 were analyzed to study immune cell infiltration in DM and AS, showcasing a histogram for the distribution of 22 leukocyte subpopulations (Figures 9A and 10A). Within the DM cohort, a notable augmentation was observed in the prevalence of several immune cells, namely B‐cells memory, T‐cells CD8, T‐cells follicular helper (Tfh), NK cells activated, monocytes, macrophages M1 and M2, along with mast cells resting. Conversely, a reduction was noted in the fractions of plasma cells, T‐cells CD4+ memory resting, NK cells resting, and mast cells activated. When contrasted with the control group, the AS cohort exhibited elevated counts of B‐cells memory, T‐cells regulatory, Tfh, T‐cells gamma delta, macrophages M0, and mast cells activated. However, the counts of B‐cells naive, plasma cells, T‐cells CD4 naive, T‐cells CD4 memory activated, NK cells resting, monocytes, dendritic cells activated, macrophages M1, macrophages M2, and mast cells resting were found to be diminished (Figures 9B and 10B). Across both diseases, an increase in B‐cells memory and Tfh were consistent, indicating their potential role in the etiology, while the counts of plasma cells and NK cells resting were consistently lower, suggesting their diminished role in disease progression. Further analysis involved Spearman correlation test to examine the relationships between pivotal genes and the identified immune cells (Figures 11A and 11B). This analysis substantiated significant correlations specifically with macrophages, T‐cells, and NK cells, underlining their potential impact on the disease mechanisms.

### 3.5 GSEA analysis

**FIGURE 12:**
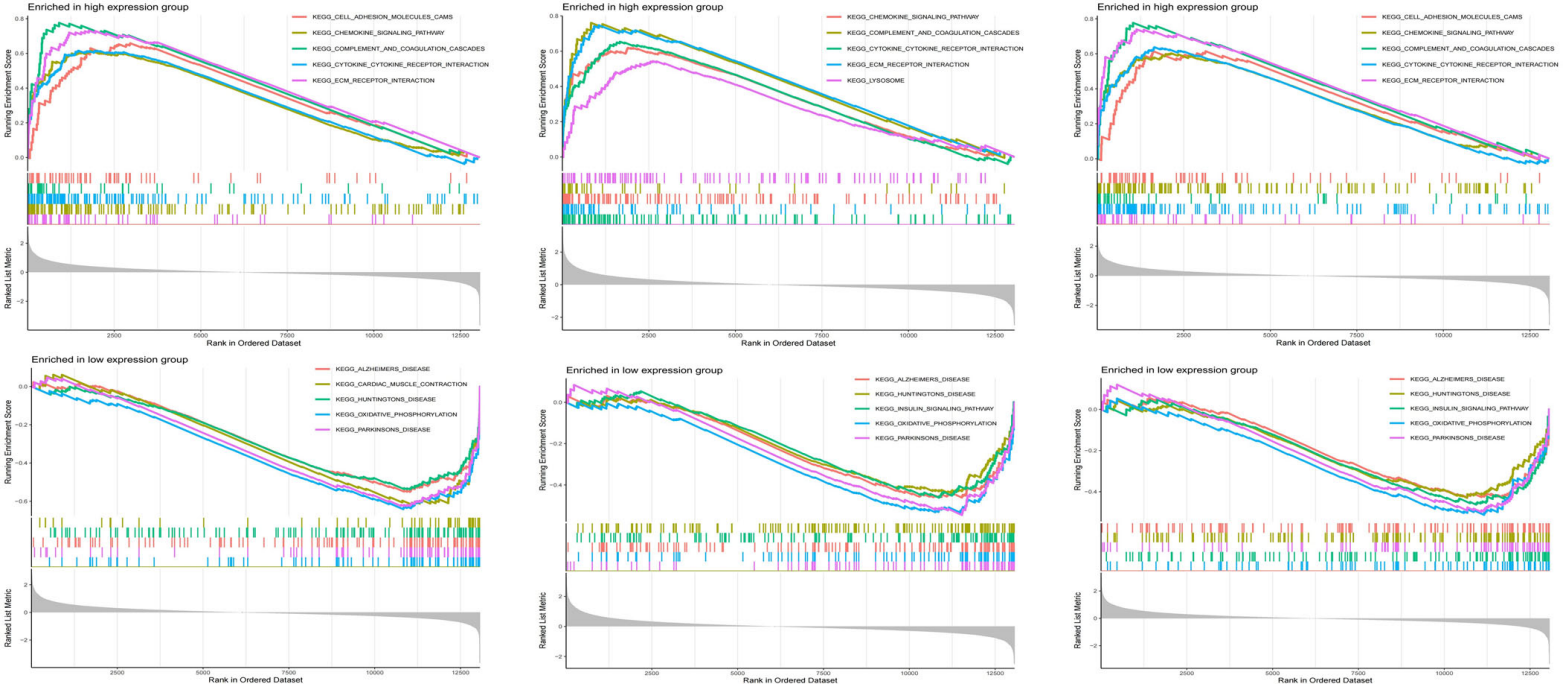
GSEA analysis of hub genes (PTPRC,TYROBP and CXCR4) in DM. DM, dermatomyositis; GSEA, Gene set enrichment analysis.

**FIGURE 13:**
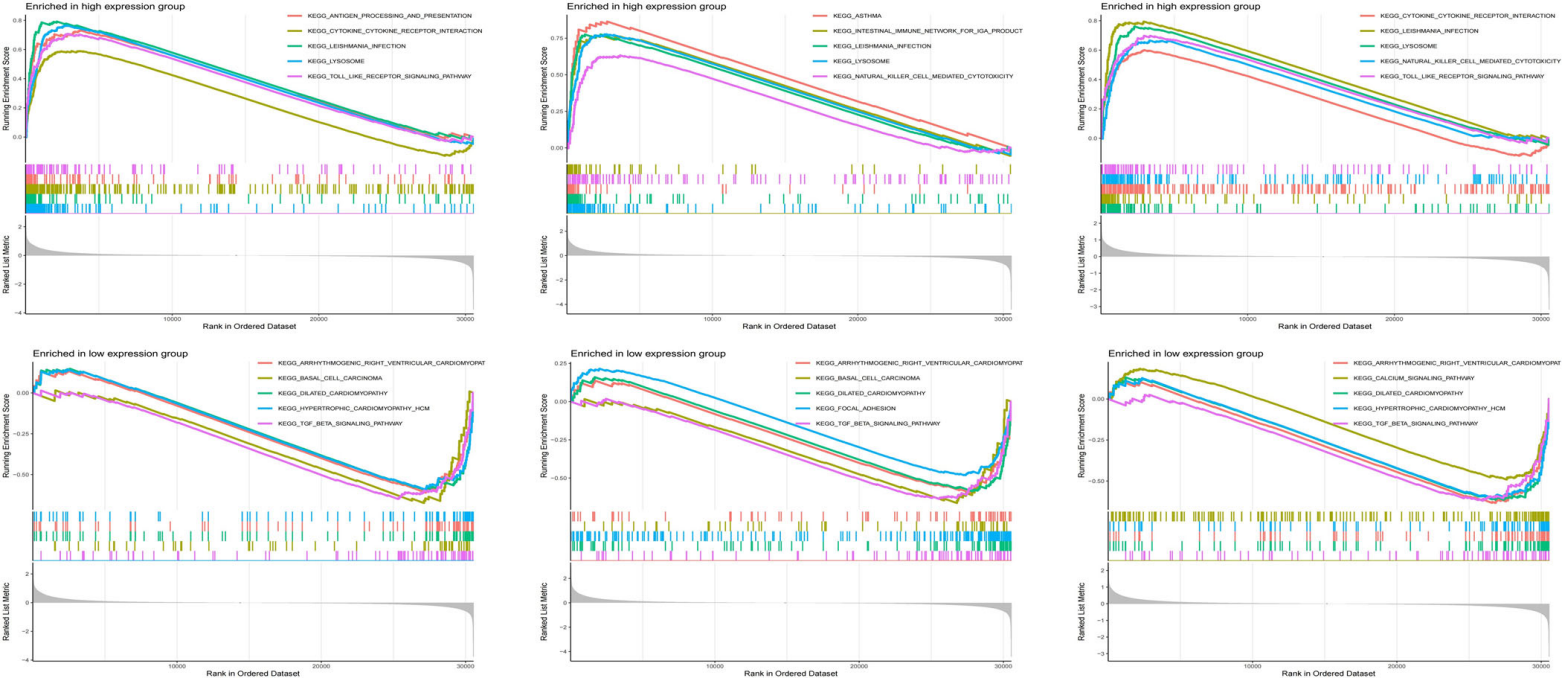
GSEA analysis of hub genes (PTPRC,TYROBP and CXCR4) in AS. AS, atherosclerosis; GSEA, Gene set enrichment analysis.

GSEA was conducted to examine the three central genes within the datasets related to DM and AS (Figures 12 and 13). Utilizing the “ClusterProfiler” package, we elucidated the five principal pathways that were either significantly upregulated or downregulated. Notably, in both conditions, these core genes were predominantly implicated in interactions with cytokine receptors and ECM (Extracellular Matrix) receptors. Furthermore, these genes were consistently found to be overrepresented across a spectrum of immune‐inflammatory pathways. This included pathways such as the chemokine signaling pathways, pathways mediated by classically activated receptors, in addition to the lysosome, complement, and coagulation cascades, highlighting their extensive involvement in the immunological responses characteristic of both DM and AS.

### 3.6 TFs prediction

**FIGURE 14:**
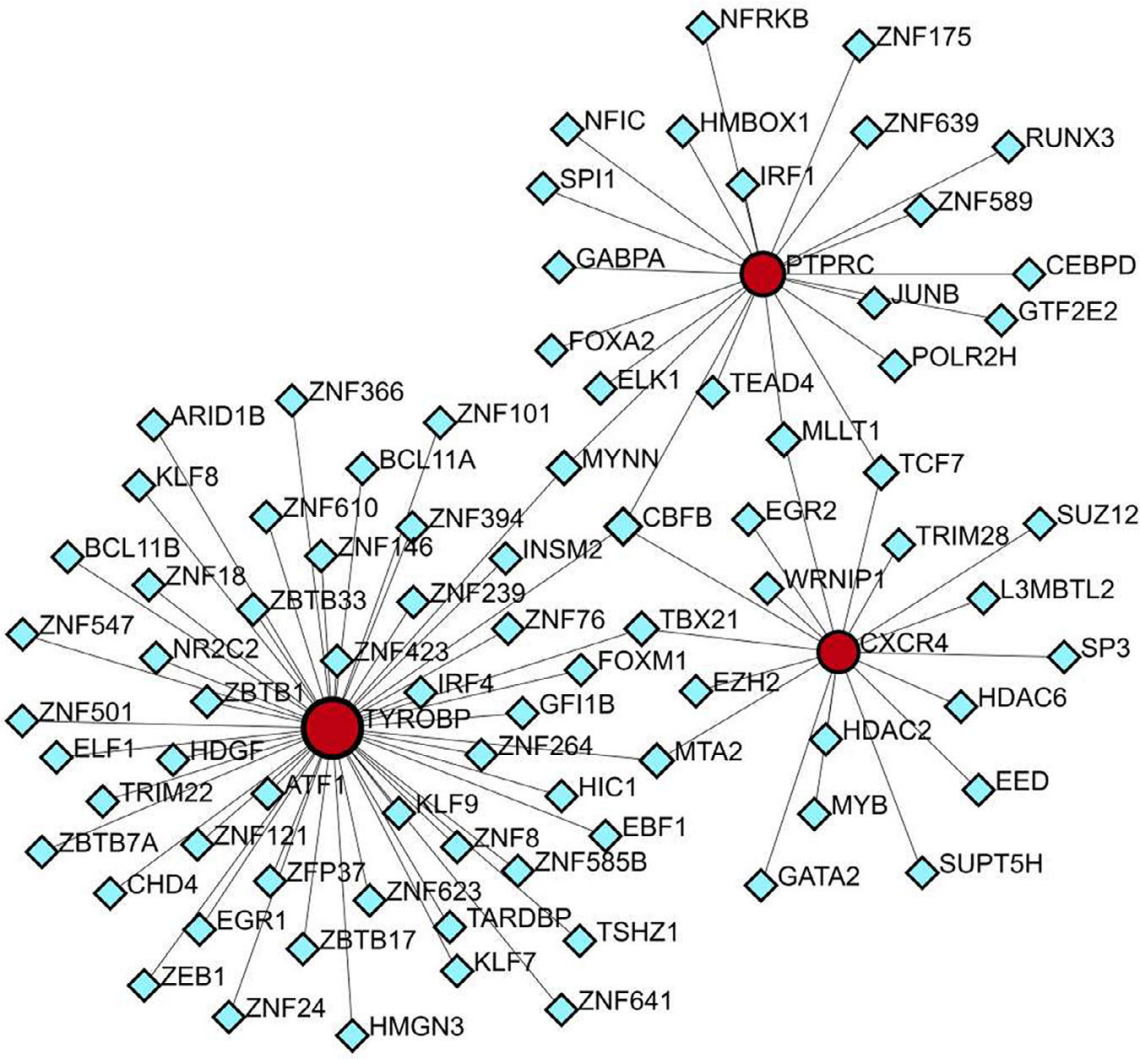
TF‐shared hub gene regulatory network. The red circle represents a shared hub gene, while the blue diamond represents transcription factors. TFs, transcription factors.

To forecast the interactions between TFs and the three pivotal genes (TYROBP, PTPRC, CXCR4), the NetworkAnalyst tool was employed to construct and visualize the TF gene regulatory network. Figure 14 demonstrates the interactions of CBFB with all three core genes, suggesting possible regulatory effects on their expression. However, further research is required to validate these findings.

## 4 DISCUSSION

Current research demonstrates that both DM and AS involve inflammatory responses and abnormal activation of the immune system. In DM and AS, the abnormal activation and infiltration of immune cells into affected tissues are common phenomena. These cells can release inflammatory factors, promoting the inflammatory process.^12^, ^15^, ^30^ Furthermore, in the immune response mechanisms against DM, T‐cells and B‐cells play a decisive role. These cells are not only key factors in the infiltration of immune cells within the inflammatory environment but also participate in the immune response to specific autoantigens by producing a series of specific autoantibodies, such as anti‐Jo‐1, anti‐Mi‐2, and anti‐MDA5 antibodies, which can directly exacerbate damage to muscles and other tissues.^31^, ^32^ Furthermore, T‐cells and B‐cells, by releasing various inflammatory mediators and cytokines such as interferon‐γ (IFN‐γ), interleukin‐6 (IL‐6), tumor necrosis factor α (TNF‐α), and interleukin‐1β (IL‐1β), not only enhance the local inflammatory response but also promote the activation and involvement of systemic immune cells. The release of these inflammatory mediators and cytokines constitutes a complex network that refines the immune system's response to myositis, encompassing both pro‐inflammatory and anti‐inflammatory processes. The imbalance of these processes is one of the key mechanisms leading to increased tissue damage and disease progression.^18^, ^33^ In the immune‐inflammatory process of AS, central mechanisms are characterized by the aggregation of immune cells along with the secretion of inflammatory agents and cytokines. Notably, immune cells, including T‐cells and B‐cells, are instrumental in the processes of infiltration and activation within AS lesions. This sequence of events culminates in the emission of inflammatory mediators like interferon‐γ (IFN‐γ), tumor necrosis factor α (TNF‐α), and a spectrum of interleukins (IL‐6, IL‐1β). These substances intensify the inflammatory reaction, thereby facilitating the genesis and progression of plaques.^34^, ^35^, ^36^ Moreover, damage to endothelial cells (ECs) and cholesterol accumulation are considered key early events in AS, triggering and exacerbating the inflammatory response of the vascular wall and prompting the accumulation and activation of immune cells such as monocytes, macrophages, and T‐cells. The transformation and activation of monocytes to macrophages occupy a central position in the development of plaques. Macrophages, by ingesting oxidized low‐density lipoprotein (ox‐LDL) and transforming into foam cells, not only exacerbate the inflammatory response but also affect the stability of plaques.^13^, ^36^ Therefore, T‐cells and B‐cells play a central role in regulating the immune response in DM and AS. Through their involvement in the production and release of inflammatory mediators and cytokines, they not only promote the formation of disease‐specific immune responses but also influence the establishment and maintenance of the inflammatory environment. This process reveals a complex regulatory network within the immune system, whose balance is crucial for maintaining tissue integrity and preventing disease progression. Understanding these mechanisms is not only vital for a deep comprehension of the pathophysiology of DM and AS but also provides possible pathways for developing new immunotherapeutic strategies against these diseases.

Endothelial dysfunction plays a key role in the pathogenesis of AS, involving the regulation of vascular hemodynamics, activation of inflammatory responses, and proliferation of vascular smooth muscle cells.^12^ ECs perform critical functions in maintaining vascular tone balance, promoting angiogenesis, participating in hemostasis, and providing antioxidative, anti‐inflammatory, and antithrombotic protection. When endothelial function is impaired, it often results in disturbed vasomotor balance, increased oxidative stress levels, exacerbated chronic inflammatory states, enhanced leukocyte adhesion and migration, and increased vascular permeability.^37^ These changes ultimately exacerbate the severity of AS. Furthermore, studies have shown that in rare systemic autoimmune diseases such as DM, specific biomarkers such as circulating endothelial cells (CECs), von Willebrand factor (vWF), endothelial progenitor cells (EPC or CEPC) and inflammatory factors are linked to endothelial dysfunction and inflammation.^38^, ^39^ These markers are related to disease activity and generally return to normal during treatment. These findings highlight the role of endothelial dysfunction in the pathogenesis of DM and suggest its potential association with increased chronic cardiovascular risk. The central role of endothelial dysfunction in AS and DM not only emphasizes the importance of endothelial health for vascular function but also reveals its potential as a biomarker for monitoring disease progression and treatment response. Therefore, a deep understanding of the molecular mechanisms of endothelial dysfunction and its role in various diseases is crucial for developing new treatment approaches and improving patient outcomes.

This study conducted a comprehensive bioinformatics analysis, exploring for the first time the common mechanisms, shared pathways, and associated immune infiltration characteristics of DM and AS. By intersecting key modular genes and common differential genes of DM and AS, three pivotal genes (TYROBP, PTPRC, and CXCR4) were identified. Further analysis of immune infiltration revealed that in the pathogenesis of these two diseases, the pivotal genes are significantly associated with macrophages, T‐cells, and NK cells. The findings from the GSEA indicate that high expression of central genes is related to receptor interactions and multiple signaling pathways.

Based on the immune infiltration atlas results for AS and DM presented in this paper, we found higher levels of B‐cells memory and Tfh helper in the disease groups. Therefore, we speculate that the pathogenesis of AS and DM shares some commonalities, which are related to B‐cells memory and Tfh.

DM, recognized as an autoimmune disorder, manifests as a multifaceted syndrome marked by the breakdown of immune tolerance.^40^ The immune system's hypersensitive reaction to antigens triggers the emergence of autoreactive T‐cells and B‐cells, alongside autoantibodies, which subsequently inflict damage on organs. Within the landscape of autoimmune diseases including DM, B‐cells assume a critical role in the disease's pathogenesis.^41^ These cells are instrumental for their dual functions: firstly, as producers of antibodies, they significantly influence the initiation and progression of immune‐mediated conditions^42^; secondly, B‐cells can serve as antigen‐presenting cells, secreting a variety of pro‐inflammatory cytokines and chemokines.^43^, ^44^, ^45^ Notably, the upregulation of the tissue B‐lymphocyte stimulator (BAFF) in muscle biopsies is pronounced, particularly in DM patients harboring anti‐Jo‐1 antibodies. Moreover, B‐cells regulatory (Bregs), a subset of cells with immunosuppressive capabilities, contribute to the maintenance of immune tolerance and homeostasis by secreting interleukins (IL)−10, IL‐15, and TGF‐β throughout the process of immune tolerance.^46^ The presence of B‐cells^47^ and terminally differentiated plasma cells^48^ within the muscle tissues of individuals with DM underscores their potential role in fostering muscular inflammation.^44^

In the context of AS, the functional specificity of B‐cell subgroups is described as diverse, encompassing both pro‐atherosclerotic and anti‐atherosclerotic properties.^49^, ^50^, ^51^, ^52^ B1a cells, identified as primary sources of naturally produced IgM antibodies, generate these antibodies without the need for antigen activation. Such antibodies are adept at recognizing oxidized lipid and apoptotic cell epitopes, playing a crucial role in mitigating the progression of atherosclerotic plaque formation.^52^ The activation process for B‐cells involves antigen attachment to B‐cell receptors (BCRs), which are specialized membrane‐bound immunoglobulins, each uniquely capable of epitope binding. These BCRs, in conjunction with other surface receptors on B‐cells like the receptor‐type tyrosine‐protein phosphatase C (PTPRC, or B220),^53^ facilitate signaling pathways essential for B‐cell activation. In hypercholesterolemic conditions, an overactivation of B‐cells can occur, leading to an imbalance in plasma cell differentiation and thereby accelerating AS development.^53^, ^54^ Therefore, the participation of B‐cells memory in the context of DM and AS demonstrates a nuanced interplay, indicating that the relationship between these immune cells and the advancement of the disease is multifaceted.

Tfh, identified as a distinct subgroup of CD4+ helper T‐cells, are crucial in facilitating the formation of germinal centers (GC), enhancing the maturation of antibody affinity, and fostering the production of B‐cells memory.^55^ Characterized by the expression of C‐X‐C chemokine receptor type 5 (CXCR5) and elevated levels of programmed death‐1 (PD‐1), these cells are pivotal in activating B‐cells through T‐cell‐dependent mechanisms, thus playing a significant role in the humoral immune response.^56^, ^57^ The secretion of immunomodulatory molecules by Tfh cells is critical for the establishment of durable B‐cell‐mediated humoral immunity,^58^ highlighting their importance in the etiology of DM and AS. Research indicates that within the GCs, interactions between Tfh and B‐cells via ICOS‐ICOSL, CD40L‐CD40, and TCR peptide‐MHC II complexes prompt Tfh cells to release IL‐4, IL‐21, and Bcl‐6.^58^, ^59^, ^60^ Interleukin‐4 (IL‐4) facilitates antigen presentation by macrophages and exhibits suppressive effects on pro‐inflammatory cytokines, contributing to the modulation of the immune response.^61^ Interleukin‐21 (IL‐21) serves a critical role across both humoral and cellular immunity, augmenting lymphocyte proliferation, enhancing the cytotoxic activity of T‐cells CD8+, improving NK cell functions, promoting immunoglobulin class switching, and fostering the differentiation of B‐cells into plasma cells.^62^, ^63^, ^64^, ^65^ These actions underscore IL‐21′s regulatory impact on the development and progression of DM and AS. Correlation analyses between these diseases and immune cell expression profiles reveal a positive association between all three central genes and at least one type of upregulated immune cell, underscoring their significance in immune response regulation. Additionally, functional annotation and co‐expression network analyses of these genes highlight their integral roles in immune system functionality and related biological processes. Based on these analyses, we hypothesize that the central genes may act as mediators in relevant immune pathways, influencing the function of immune cells.

PTPRC, commonly referred to as CD45, is a receptor‐type protein tyrosine phosphatase that is highly conserved throughout evolution. Its expression is prevalent across almost all cells of hematopoietic origin, with the exception of mature erythrocytes, playing a pivotal role in regulating antigen receptor‐mediated activation in T‐ and B‐cells, thus significantly influencing the innate immune system.^66^, ^67^ Investigations have elucidated the elevated expression levels of PTPRC in tissues obtained from patients diagnosed with DM.^68^ By regulating lymphocyte survival, cytokine responses, and TCR signaling, PTPRC controls immune function,^69^ and is under investigation as a therapeutic target for various immune‐related conditions, including autoimmune diseases and organ transplantation.^67^ Nonetheless, a definitive association between PTPRC expression and DM progression remains to be established. In the context of AS, PTPRC's role as a central gene has been acknowledged,^69^, ^70^, ^71^, ^72^, ^73^ particularly regarding its involvement in the disease's advancement through mechanisms linked to T‐cells regulatory,^74^ a correlation further substantiated by experimental models in animals.^75^ Hence, the augmented expression of PTPRC emerges as a critical factor for both DM and AS, underscoring the therapeutic potential of PTPRC inhibitors in mitigating the progression of these diseases.

TYROBP, also recognized by the designations DAP12 and KARAP, is identified as a transmembrane adaptor protein pivotal in constituting part of the activation subunits associated with NK cells' receptors. This protein manifests across a diverse array of cell types, inclusive of peripheral blood mononuclear cells, macrophages, dendritic cells, and osteoclasts. The essential role of TYROBP in supporting the functionality of an array of receptors located on the microglia's plasma membrane has recently garnered concentrated scholarly interest.^76^ It has been substantiated through various studies that TYROBP's deficiency significantly influences microglial polarity from a molecular perspective in initial phases, and consequentially modifies the microglial activation timeframe as well as the destiny of motor neurons during neurodegenerative processes in subsequent phases.^77^, ^78^

Activation of microglia triggers the secretion of mediators with pro‐inflammatory and anti‐inflammatory properties, hence playing a multifaceted role in the pathology of central nervous system injuries.^79^ Presence of infections, tissue damages, or stimulatory signals within the microenvironment prompts microglia activation. This activation leads to both phenotypic and morphological alterations, propelling these cells towards the site of damage or stimulation. Consequently, this movement sets off an inflammatory response. In their activated form, microglia, classified into M1/M2 phenotypes, release neurochemicals which can provide neuroprotection or cause neurotoxicity.^80^, ^81^ The M1 phenotype distinguishes itself through the production of pro‐inflammatory cytokines (for instance, TNF‐α, IL‐6, IL‐1β), chemokines, and reactive oxygen species (ROS), playing a pivotal role in initiating acute immune responses. In contrast, the M2 phenotype is primarily involved in producing anti‐inflammatory cytokines (such as IL‐4, IL‐13), playing a key role in tissue regeneration, removal of debris, wound healing, and restoring equilibrium in brain function.^82^ As critical elements of the mononuclear phagocyte system, microglia also undertake macrophage‐like duties, which include the detection and surveillance of dead cells, pathogens, and various substances, both internal and external.^83^ This delineates TYROBP's significant influence on microglial activation, thereby underscoring its regulatory capacity in immunological‐inflammatory conditions such as DM and AS. This assertion aligns with our observations and is further corroborated by Liu et al.,^84^ who, through prior bioinformatic examination, validated TYROBP's implication in AS progression. Consequently, targeting TYROBP therapeutically emerges as a feasible approach to ameliorate DM and AS conditions.

CXCR4 is a widely expressed 7‐transmembrane G‐protein coupled receptor,^85^, ^86^ binding to its ligand CXCL12, inducing intracellular signaling through typical heterotrimeric G‐proteins.^87^ The G proteins activated by CXCR4 include Gai1, Gai2, Gai3, Gaq, and Gao, with Gai1 and Gai2 subtypes being the most efficient.^88^ Research by Lv et al.^89^ demonstrated using immunohistochemistry, the preferential expression of CXCR4 in vascular perimysial inflammatory infiltrates in DM. Western blot analysis revealed significantly upregulated expression of CXCR4 in the muscle homogenates of DM patients compared to healthy controls.

Over recent decades, the essential function of Type I interferons (IFN‐I), specifically interferon‐α (IFN‐α) and interferon‐β (IFN‐β), in the etiology of DM and other autoimmune maladies has been well documented.^90^, ^91^ Notably, plasmacytoid dendritic cells (pDCs) possess the capability to generate IFN‐α at levels surpassing other cell types by over a thousandfold, earning them the designation as natural IFN‐α producing cells (NIPCs).^92^ Consequently, the unusual gathering of pDCs within the perimysium and endomysium is recognized as a key intramuscular origin of IFN‐I in Diabetes Mellitus (DM). Research by Lv et al.^89^ has elucidated the pivotal role of chemokine receptors, including CXCR3, CXCR4, and CCR7, in directing the transit of pDCs from the peripheral bloodstream to muscular tissue in subjects with DM via diverse mechanisms. In detail, the recruitment of pDCs into follicle‐like cellular aggregations in DM is effected through the interaction of CXCR4 and CCR7 with high endothelial venules (HEVs). Contrary to CXCR3, it has been observed that CXCR4 and CCR7 uniquely support the continual relocation of pDCs, even in the absence of inflammatory stimuli.^93^, ^94^, ^95^ This suggests their involvement in the regulation of DM pathology.

In the etiology of AS, the chemokine receptor CXCR4 is notably present across a diverse range of cellular entities implicated in CVD, encompassing ECs, Vascular Smooth Muscle Cells (VSMCs), T‐cells, B‐cells, neutrophils, monocytes, and macrophages.^71^ Investigations spearheaded by Merckelbach et al.^96^ have illuminated a marked upsurge in the transcriptional and protein concentrations of CXCR4 and CXCL12 within carotid atherosclerotic plaques, as contrasted with normative vascular structures.^97^, ^98^ Further substantiation from zoological research delineates CXCR4's contributory role in AS, evidencing that its augmented expression within murine cardiac tissue amplifies infarct dimensions, diminishes myocardial efficiency, and catalyzes the influx of pro‐inflammatory cellular units. Concurrently, hypoxia emerges as a pivotal factor in AS progression, principally through the inducement of CXCR4 expression via hypoxia‐inducible factor 1 (HIF1) activation, thereby facilitating cellular migration in the direction of CXCL12.^99^, ^100^ The interference with CXCR4's function, particularly in the contexts of aging, apoptotic processes, and the systemic purging of neutrophils, has been implicated in the exacerbation of AS owing to enhanced cellular adhesion and migration.^98^ It is critical to acknowledge that CXCL12 is not the exclusive ligand for CXCR4, indicating the existence of additional regulatory modalities for CXCR4 stimulation. Notably, the Macrophage Migration Inhibitory Factor is capable of binding to and activating the CXCR4 pathway, consequently exerting a pronounced atherogenic influence.^101^ Moreover, Zernecke et al.^102^ have elucidated that miRNA126 enhances CXCL12 synthesis by instigating an autocrine loop of CXCR4 signaling, culminating in the attenuation of AS and ensuring vascular safeguarding dependent on CXCL12. Thus, emerging evidence posits CXCR4 as a viable biomarker for discerning individuals afflicted with DM and AS.

This manuscript underscores the innovative utilization of bioinformatic approaches to decipher the overlapping molecular mechanisms and pathways implicated in both DM and AS, spotlighting crucial genes and the characteristics of immune infiltration pertinent to both conditions. These findings could further elucidate the shared mechanisms between DM and AS. Nevertheless, it is pertinent to acknowledge the constraints associated with our analysis. Fortunately, the diagnostic model based on three key genes also proved effective in an external validation set, partially enhancing the credibility of our results. Chiefly, the investigation adopts a retrospective design, necessitating the corroboration of our findings through prospective experimental and clinical investigations. Chiefly, the investigation adopts a retrospective design, necessitating the corroboration of our findings through prospective experimental and clinical investigations. Furthermore, it is critical to assert that the diagnosis of DM and AS should not be overly reliant on the detection of these shared genetic and pathway markers alone, but should also incorporate conventional methods such as invasive biopsies and histopathological examination for definitive confirmation. Despite these caveats, our research potentially contributes novel insights and biomarkers for understanding the molecular interplay between DM and AS. The integration of these genetic markers with other clinical diagnostic frameworks and their targeted exploration holds substantial promise for advancing the diagnostic and therapeutic strategies for these diseases.

## 5 CONCLUSION

In this research, three genes that are commonly expressed (PTPRC, TYROBP, and CXCR4), along with pathways that are regulated together and similar immunological characteristics, were identified for DM and AS. This identification led to the creation of a diagnostic model that is efficacious. The investigation uncovers a plethora of pathogenic mechanisms that DM and AS share, pointing towards a significant overlap in their disease etiologies. The analysis conducted provides novel viewpoints on the concurrent molecular mechanisms underlying DM and AS, encompassing genetics, signal transduction pathways, and immune cell infiltration.

## CONFLICT OF INTEREST STATEMENET

The declaration by the authors confirms that the study was performed without the influence of any business or financial affiliations that might be perceived as a conflict of interest.

## ETHICAL APPROVAL

This study does not contain any studies with human participants or animals performed by any of the authors.

## Supporting information

Supporting Information

Supporting Information

Supporting Information

Supporting Information

## Data Availability

The datasets used in this study are publicly available in the NCBI Gene Expression Omnibus (GEO) database, with the accession numbers including: GSE1551, GSE128470, GSE143323, GSE28829, GSE100927, and GSE43292. The data supporting the findings of this study are available from the corresponding author upon a reasonable request.
