# Supplementary figures and images for: Identification of common mechanisms and biomarkers for dermatomyositis and atherosclerosis based on bioinformatics analysis
Source: Skin Res Technol. 2024 Jun 20;30(6):e13808. doi: 10.1111/srt.13808 (PMC11187814; doi:10.1111/srt.13808)

Scale Free Topology Model Fit, signed  $R^2$

**Scale independence**

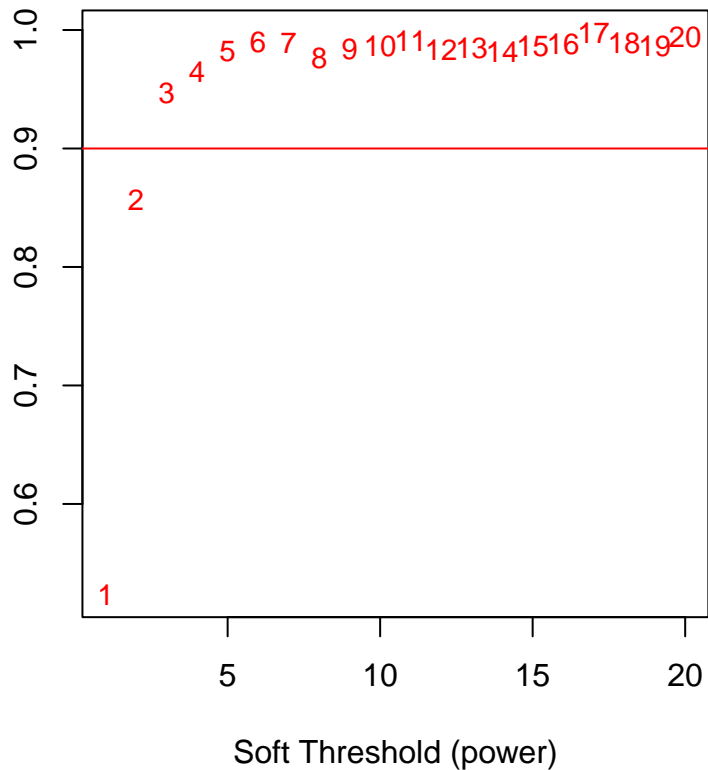

**Mean connectivity**

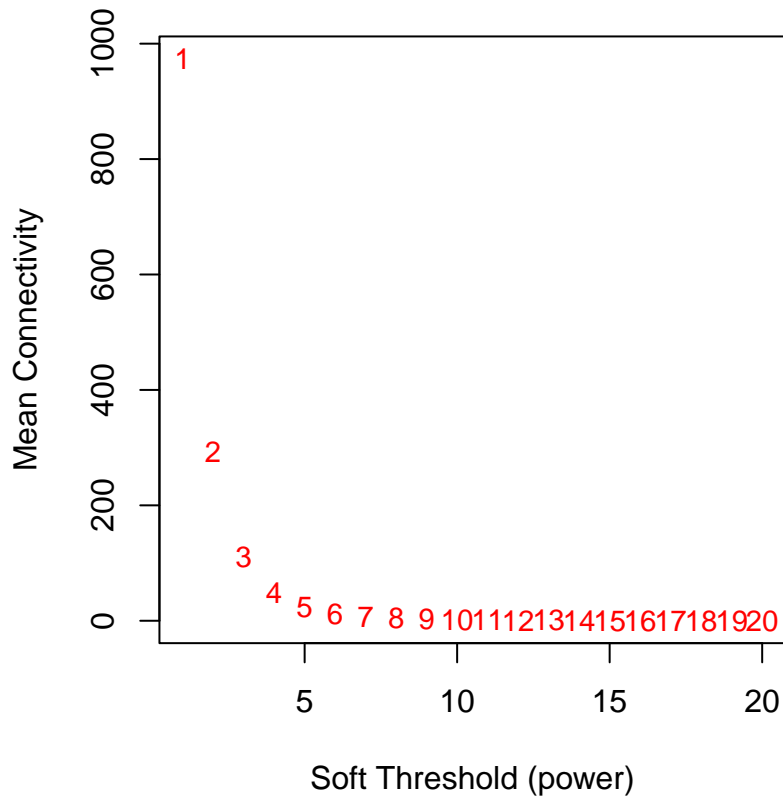

Supplement: Supplementary file 1 — Supporting Information [file SRT-30-e13808-s002.pdf]

### Scale independence

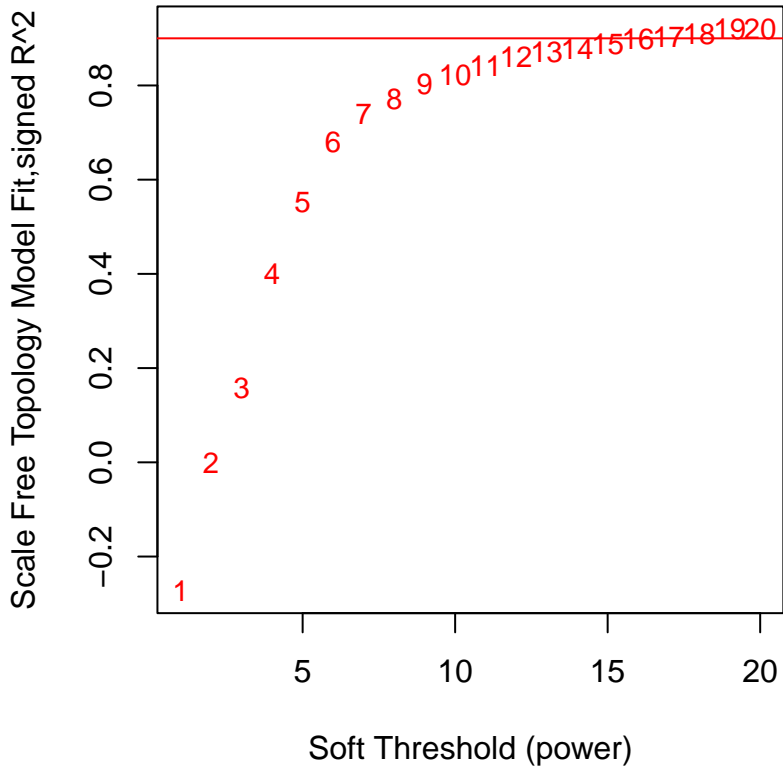

### Mean connectivity

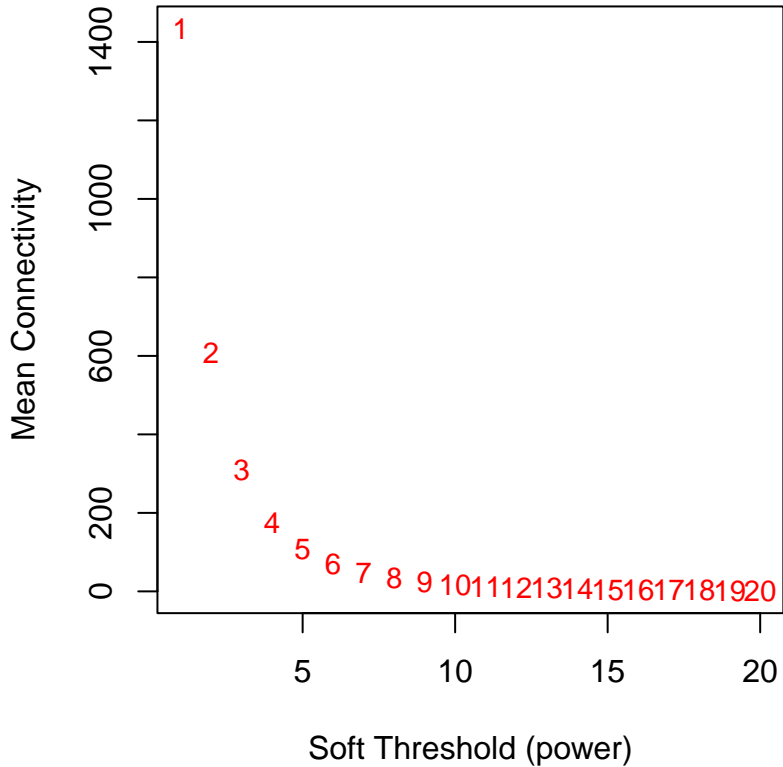

Supplement: Supplementary file 2 — Supporting Information [file SRT-30-e13808-s004.pdf]

**Module membership vs. gene significance**  
**cor=0.76,  $p < 1e-200$**

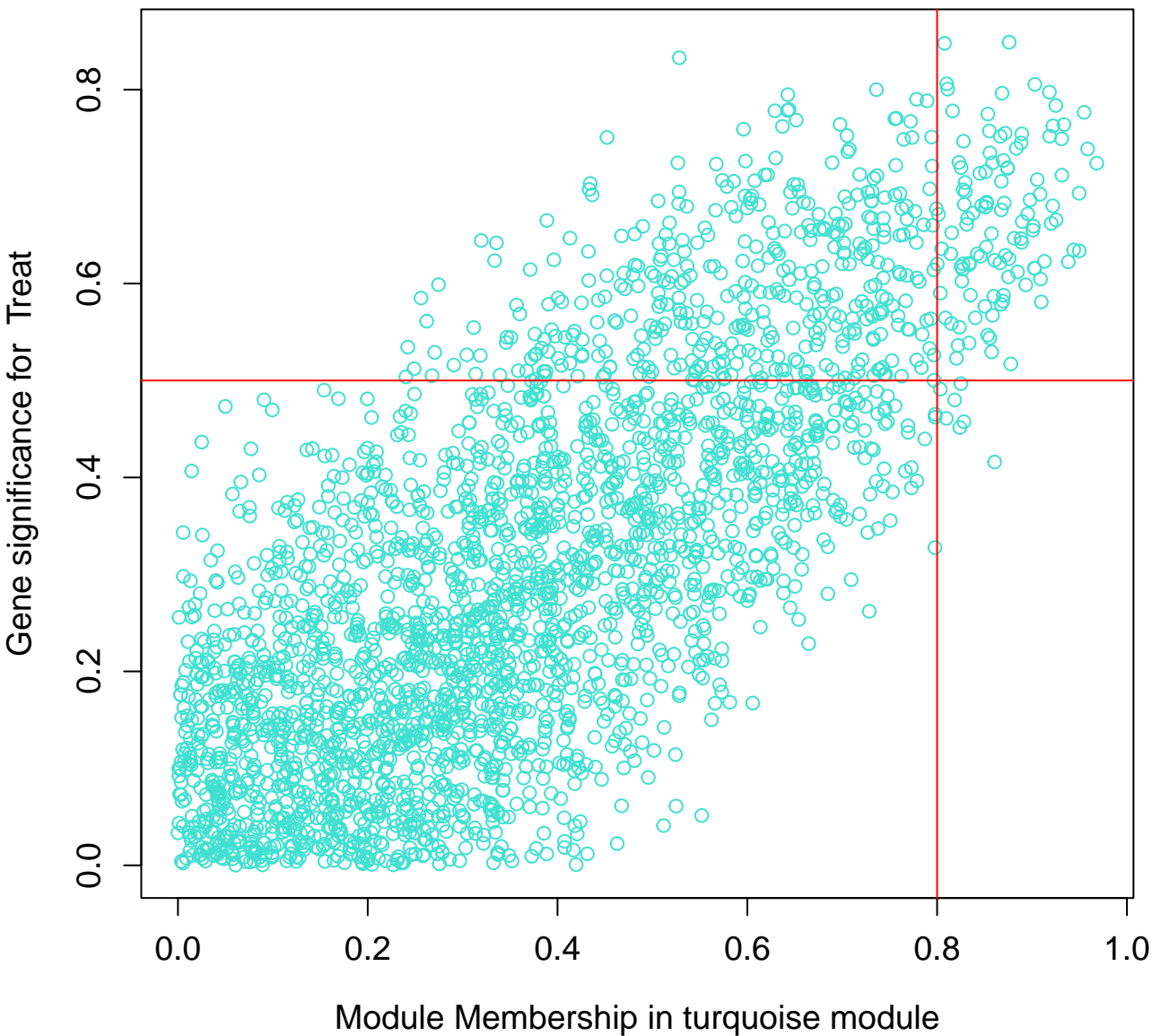

Supplement: Supplementary file 3 — Supporting Information [file SRT-30-e13808-s003.pdf]

**Module membership vs. gene significance**  
**cor=0.75,  $p < 1e-200$**

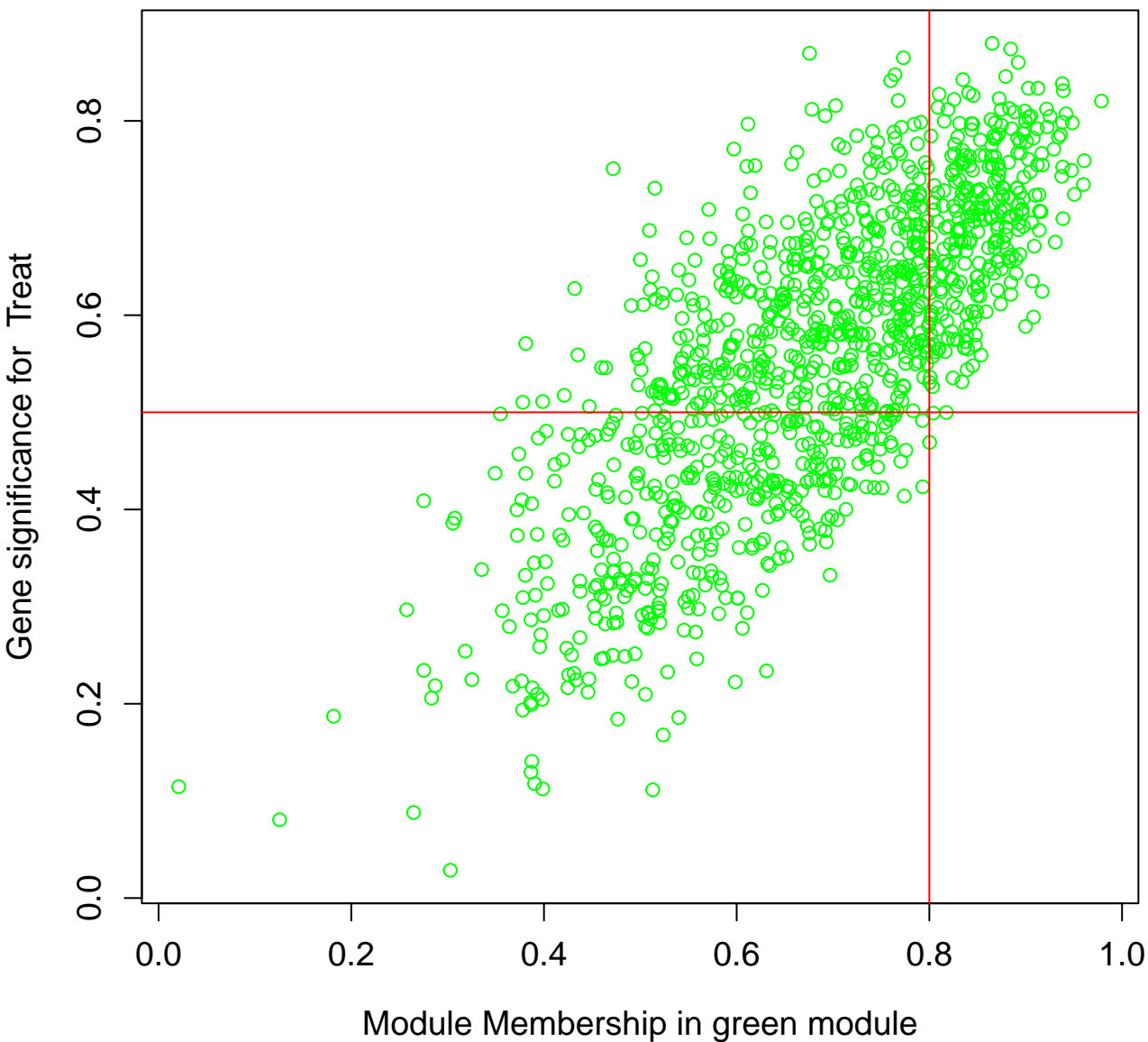

Supplement: Supplementary file 4 — Supporting Information [file SRT-30-e13808-s001.pdf]
